# Diet and Neurodevelopmental Score in a Sample of One-Year-Old Children—A Cross-Sectional Study

**DOI:** 10.3390/nu11071676

**Published:** 2019-07-21

**Authors:** Eli Anne Myrvoll Blomkvist, Elisabet Rudjord Hillesund, Sissel Heidi Helland, Indra Simhan, Nina Cecilie Øverby

**Affiliations:** 1 Faculty of Health and Sport Sciences, Department of Public Health, Sport and Nutrition, University of Agder, 4630 Kristiansand, Norway; 2 Department of Child and Adolescent Mental Health, Sorlandet Hospital, 4615 Kristiansand, Norway

**Keywords:** neurodevelopment, ages and stages questionnaire (ASQ), children, diet, fruits and vegetables, vegetable intake, fish intake, dietary factors, breastfeeding

## Abstract

Environmental factors in the first years of life are crucial for a child’s neurodevelopment. Research on the association between breastfeeding and neurodevelopment is inconclusive, while research on the possible association between other dietary factors and neurodevelopment is inadequate in children as young as one year of age. The aim of the present study was to investigate associations between both breastfeeding and other dietary factors and the neurodevelopment of one-year-old children in Norway. Methods: Participants were recruited from kindergartens in four Norwegian counties in 2017. A questionnaire including questions about dietary factors and breastfeeding, and a standardised age-related questionnaire on neurodevelopment (the Ages and Stages Questionnaire), were completed by parents of one-year-olds. Linear regressions adjusting for relevant covariates were conducted to explore the associations. Results: In our sample of 212 one-year-old children, a longer duration of breastfeeding was associated with higher neurodevelopmental scores. Dietary intake of fish, fruits and vegetables was also strongly associated with higher neurodevelopmental scores, even after adjustment for breastfeeding and maternal education. Conclusion: Our results indicate that healthy dietary factors are important for neurodevelopment in young children, with measurable effects already at the age of one year.

## 1. Introduction

The first years of life are crucial to a child’s neurodevelopment. Neurodevelopment concerns the acquisition of skills in a variety of developmental domains, including fine and gross motor function, language and social adaptation skills and cognition. Early neurodevelopment tracks into later in life and is important for later IQ and academic achievement [1,2,3,4]. Genetic, biological and environmental factors such as sex, gestational age, maternal mental health, maternal education and parental socio-economic status are all factors that can influence neurodevelopment [1,5,6].

Nutrition also influences neurodevelopment and adequate diet quality is therefore of utmost importance in the early years in which brain development is at its peak. Infants’ nutrient requirements are high in order to meet the demands of their growth and development.

The World Health Organization (WHO) recommends exclusive breastfeeding for at least 6 months, while the European Society of Paediatric Gastroenterology, Hepatology and Nutrition Committee on Nutrition (ESPGHAN), as well as the Norwegian Health Directorate, recommends the introduction of complementary foods between 4–6 months of age [7,8,9].

Longer duration of breastfeeding has been associated with better cognitive and motor development in preschool children [10,11,12,13,14]. Although several studies have demonstrated an association between breastfeeding and cognitive functioning, results from observational studies are diverse, especially after adjusting for possible confounders such as socioeconomic status and maternal IQ [15,16,17].

Observational studies suggest that several micronutrients, including omega-3 fatty acids, zinc, iron and iodine play important roles in children’s brain development [18]. Since nutrients do not act alone, and individuals consume combinations of food, Nyaradi et al., suggest that public health interventions should focus on the promotion of overall diet quality rather than isolated micronutrients [18]. 

Healthy dietary patterns in childhood can influence later cognitive and neuropsychological outcomes [19,20]. A recent review found a positive association between healthier foods (wholegrains, fish, fruits and/or vegetables) and executive functioning in children and adolescents, whereas less healthy snack foods, sugar-sweetened beverages and red/processed meats were inversely associated with executive functioning [21]. Smithers et al. found that healthier dietary patterns from 6 to 24 months may have a small but persistent effect on IQ at 8 years [19]. Nyaradi et al. found that a high-quality diet in the early years had a positive effect on academic achievement at ages 10 and 12 [3]. However, to our knowledge, no studies have so far investigated the association between diet and child neurodevelopment measured by standardised screening tools in one-year-olds. With this present study, we aimed to investigate whether breastfeeding and the intake of selected foods and food groups in infancy are associated with higher neurodevelopmental scores as early as at the age of one year.

## 2. Materials and Methods

### 2.1. Study Design and Participants

Data for the present study were derived from the baseline study of a web-based cluster randomised controlled trial among one-year-old children in kindergartens in Norway. The study protocol for this trial has been published elsewhere [22]. The protocol was prospectively registered in the International Standard Randomised Controlled Trial Number registry (reg.nr. ISRCTN98064772) and approved by the Norwegian Centre for Research Data (ref.nr. 49951). 

The recruitment of kindergartens started in May 2017 and targeted all public and private kindergartens in four Norwegian counties (Telemark, Oppland, Sør-Trøndelag and Møre og Romsdal) that met the inclusion criterion (*n* = 1043): having children born in 2016. Before randomisation, the pedagogical leaders in participating kindergarten departments were asked to distribute an electronic invitation letter to the parents of children born in 2016 that provided detailed information about the study and a link to the registration web page where parents could register their child to the study. Parents were informed that they consented to participate by registering their child. Inclusion criteria for child enrolment were that they had to be born in the year of 2016 and that at least one of the parents had to be able to read and understand Norwegian. Parents could register their child for the study from late August 2017 until the end of October, two weeks before the intervention started in the kindergartens in November 2017. In total, 267 children were registered for the study. The baseline questionnaires were sent to parents by e-mail shortly after registration and had to be completed electronically before randomisation. 

### 2.2. Measures

Primary and secondary outcomes of the randomised controlled trial, as well as all measures and instruments, are presented in the study protocol [22]. Parents completed a comprehensive questionnaire including all the outcome variables, except measures of neurodevelopment which were measured with the Ages and Stages Questionnaire (ASQ) [23]. Both questionnaires were administered as online surveys via links sent to the parents’ e-mail addresses shortly after registering their child for the study. Measures relevant to the outcomes in the present paper are described below. 

#### 2.2.1. Measures of Child Neurodevelopment

Children’s neurodevelopment was measured with the Ages and Stages Questionnaire [23]. ASQ is a developmental assessment tool kit for parents who complete the questionnaire at prescribed intervals, covering the age-range of 4 to 60 months. Each questionnaire consists of 30 described and illustrated questions divided into five different domains: communication/language, gross motor, fine motor, problem-solving and personal-social skills. The scoring is “yes” (10 points), “sometimes” (five points) and “not yet” (0 points), depending on the question whether the child has a certain skill or behaviour. While completing the questionnaire, the child has to be together with the parent to try out certain tasks or activities (e.g., Does your child stack a small block or toy on top of another one? and After you have shown your child how, does he/she try to get a small toy that is slightly out of reach by using a spoon, stick, or similar tool?). The maximum score is 60 points per domain, i.e., a total of 300 points maximum. The ASQ has been shown to be cost-effective, easy to use, appreciated by parents and has been widely used in both clinical and research settings in several countries [24,25,26]. The Norwegian version of ASQ has been validated and compared with US normative data [5,27]. The ASQ was made electronic by transferring questions and their corresponding illustrations to a web-based survey. When administering the electronic version of these questionnaires, we had no access to information on whether the child was born to term or not. The ASQ was therefore administered according to date of birth, and hence chronological age in months. The ASQ includes the question of whether the child was born to term, and if not born to term, how many weeks prior to term. As children born more than 3 weeks before term should have completed an age-adjusted questionnaire, children born before 37 completed pregnancy weeks (*n* = 12) were excluded from the analysis.

#### 2.2.2. Dietary Intake

Child food intake was measured by selected items from a food frequency questionnaire (FFQ) that has been validated and used in large national surveys of the food habits of children ages one- and two-years-old in Norway [28,29]. The frequency of intake was assessed without specification of the amounts consumed. Questions on how often the child eats a broad selection of vegetables (for example “carrots”, or vegetable categories, such as “onions and leek”) and fruits (for example “bananas”, or fruit categories, like “oranges, clementines and such”) were included, in addition to questions about berries, potatoes, pasta and rice, bread and cereals, spreads, drinks, warm meals, sweets and snacks. All frequencies of intake were re-coded into times per week. The response options for intake of fruits and vegetables and how they were re-coded into times per week were as follows: never = 0, <1/month = 0.1, 1–3/month = 0.5, 1–2/week = 1.5, 3–4/week = 3.5, 5–6/week = 5.5, 1/day = 7, 2/day = 14, >3/day = 21. 

Breastfeeding was assessed with a question of whether the child was still breastfed, and if he or she was still breastfed, how many times per day and night. The duration of breastfeeding was assessed with a question of the child’s age when he or she stopped receiving breast milk, measured in weeks from birth to 4 weeks, and in months from 2 to 12 months and further. The duration of breastfeeding was re-coded into weeks.

#### 2.2.3. Other Baseline Measures

Parents were asked to provide their child’s date of birth, gender, whether the child was born in Norway, and weight and length at 12 months of age as recorded in the child’s health card or weight and length from the most recent health control if the child was under the age of 12 months. Children’s gender and date of birth were also checked against the registered data on the registration web page.

#### 2.2.4. Measures of Parents’ Socio-Demographics

Parents’ marital status was assessed, entailing six response options: single, married, cohabiting, separated, divorced or other. The study asked for the highest completed education of both parents with five response alternatives: less than 9 or 10 years of primary school, primary school, secondary school or high school, university 4 years or less or university more than 4 years. The work situation of the one parent who answered the questionnaire was assessed with the following response alternatives: work full-time, work part-time, “housewife”, sick leave, leave, disabled, occupational rehabilitation, student, unemployed or other work situation. In addition, parents entered their own age in years, and the parent completing the questionnaire entered his or her gender. The non-Norwegian descent of both parents was approximated by the question of whether they were born in Norway. Parents also reported their own weight in kilograms and height in centimetres. 

### 2.3. Statistical Analysis

Data analysis was performed using SPSS Statistics, version 25.0. Baseline characteristics expressed as mean and standard deviations (SD) and median and proportions as appropriate, were explored using descriptive statistics. 

For the analyses, we combined the intake of some food groups to represent new variables: 

1. Fresh fruits, vegetables, fresh potatoes, legumes and unsalted nuts into All fruits and vegetables.

2. Lean fish, fatty fish, fish products (processed fish), fish spread and roe spread into Fish and fish products.

3. Unprocessed red meat, unprocessed white meat (poultry), minced meat, sausages, liver paste (spread made of pork liver), ham and cold cuts into All meat and meat products.

4. Wholegrain bread, wholegrain crispbread, oatmeal porridge, oatmeal and müsli into Wholegrain products.

5. Sweets and candy, salted snacks, ice cream, biscuits (both sweet and salty), sugary drinks, sweet pastries and chocolate spread into Typical sugary foods.

Associations between ASQ-scores and dietary factors were explored using crude and multivariable linear regression. Both ASQ-scores and the included dietary factors were inspected for deviations from normality. Since the mean ASQ total score has been shown to differ slightly between age groups, we first tested the associations adjusting only for child age, but this did not change the estimates. Based on the literature regarding potential influences on ASQ score, we entered the child’s age, gender and parents’ education as covariates in a multiple regression model. Marital status and ethnicity were not considered relevant because most of the parents were cohabitant and Norwegian-born. In addition, we fitted a model where breastfeeding was included as a covariate together with the above-mentioned covariates. We also performed a robustness test by doing the regression analysis excluding those who had never been breastfed (*n* = 15) to check whether the associations remained the same.

## 3. Results

### 3.1. Study Sample

Table 1 presents the baseline characteristics of the study population. Out of the 267 children registered for the study, 246 parents answered the main questionnaire on demographics, dietary behaviour and other outcomes. Among the children included, all were born in Norway; 47.6% were girls, and the children’s mean age was 16.7 (3.0) months. Among the parents who completed the questionnaire, 88.6% were women. The parents mean age was 31.2 years (SD 4.7), and 90.5% were born in Norway. Most parents were living together (94.3%), and 63.8% of the mothers and 42.3% of the fathers had higher education (University or College). 

### 3.2. ASQ

The ASQ was completed by 232 parents. The number of age-appropriate ASQ forms distributed, number of completed questionnaires in each age group, ASQ scores in mean (SD) and median (min-max) are presented in Table 2. The mean ASQ total score across all age-in-months groups was 235.3 (SD 37.4). 

### 3.3. Dietary Intake

The children’s frequency of intake of relevant foods and food groups are presented as times per week in Table 3. The mean intake of vegetables was 20.3 times per week (SD 11.3); fresh fruits 17.0 times per week (SD 10.7) and that of all fruits and vegetables was 40.5 times per week (SD 19.9). The mean intake on lean and fatty fish was 2.1 times per week (SD 1.3) and that of total fish and fish products including fish as a spread was 5.9 times per week (SD 3.7). The mean intake of unprocessed meat was 2.2 times per week (SD 1.8), while that of all meat and meat products combined (including processed meats and spread) was 10.1 times per week (SD 4.4). The mean intake of wholegrain products was 11.7 times per week (SD 5.0) and that of typical sugary foods was 3.9 times per week (SD 3.7). The mean duration of breastfeeding was 33.3 weeks (SD 18.4), corresponding to approximately 8 months (Table 1). 

### 3.4. Associations Between Dietary Factors and ASQ Score

A total of 212 children, i.e., those born to term with completed questionnaires on both dietary factors and ASQ score, were included in the main analyses (Table 4). 

The ASQ total score was significantly associated with the duration of breastfeeding (β 0.42, *p* = 0.004). The ASQ score increased with 0.4 points for every additional week of breastfeeding. The frequency of intake (times/week) of vegetables, fresh fruits and berries, fish and all fish products, respectively, was also positively associated with ASQ score (Table 4). Adjustment for potential confounders only resulted in minor attenuation of estimates and significance level. In an additional regression model, we adjusted for the duration of breastfeeding as a potential confounder of the associations between healthy dietary items and child development. The associations between the ASQ total score and the selected dietary factors attenuated slightly but remained significant after adjusting for breastfeeding. The strongest associations with the ASQ score in the fully adjusted model was observed for fish intake (β 3.90, *p* = 0.049) and intake of vegetables (β 0.88, *p* < 0.001). An increase of one serving of fish per week translated into a four-point higher ASQ score. An additional serving of vegetables per day translated into a six-point higher ASQ score (0.88/week × 7 days = 6.16). 

Since there was a positive association between all meat products combined and ASQ, while no such association was found for unprocessed meat, we explored the “all meat” variable to see if there were differences between different meat types and meat products. We found that the intake of liver paste (mean 3.8 times per week, SD 2.2) was strongly associated with ASQ total score (β 2.29, *p* = 0.044), while other types of meat or meat products did not result in any association with ASQ score.

Wholegrain products were positively associated with ASQ in both the unadjusted and adjusted model, but when we included breastfeeding as a covariate in the third model, this association was no longer present. No association was observed between ASQ total score and combined intake of typically sugary foods in this sample of children (table 4). 

As a robustness test, the adjusted regression analyses were repeated in a sample, (*n* = 197), where those who had never been breastfed were excluded (*n* = 15). All effect sizes remained practically the same, however, the significance level changed slightly for the associations regarding fruits and berries (*p* = 0.059), lean and fatty fish (*p* = 0.071) and liver paste (*p* = 0.056).

## 4. Discussion

In the present study, we aimed to examine potential associations between frequency of intake of selected foods and neurodevelopment as measured by ASQ total score in a sample of Norwegian one-year-olds. To our knowledge, there are few, if any, reports on whether and how dietary factors other than breastfeeding are associated with neurodevelopment in children this young. We found that the neurodevelopmental score was not only positively associated with duration of breastfeeding, but also positively associated with the frequency of the children’s intake of fresh fruits, berries and vegetables, and with fish and fish products. 

There are few studies comparable to the present study, both including other dietary factors than breastfeeding, and evaluating early diet and neurodevelopment in children as young as one to two years. We found that breastfeeding duration was associated with higher total neurodevelopmental scores. For this relation, previous results have been conflicting. Several studies have found similar associations, like the EDEN study of more than 1000 children [11]. They observed a positive association between longer breastfeeding duration and better cognitive and motor development, measured with the ASQ in 2- and 3-years-old children. Likewise, the Rhea study from Greece and the Mother’s and Children’s environmental Health (MOCEH) study from Korea showed similar results [10,13]. In these two studies, neurodevelopment was assessed by using the Bayley Scales of Infant and Toddler Development. There is, however, some controversy in this regard, with Holme et al., 2010 and Boutwell et al., 2012 showing no associations and arguing that sociodemographic factors and maternal IQ can explain the association between breastfeeding and neurodevelopment/cognitive development [17,30]. In our study, maternal education was adjusted for. One mechanism that could explain breastmilk’s relation to neurodevelopment is that breastmilk provides the nutrients required for brain development, such as lipids, complex proteins and carbohydrates, as well as vitamins, minerals and other biologically active components [31,32]. In addition, it is suggested that the physical and socioemotional contact between mother and child during breastfeeding can influence neurodevelopment [33]. 

Beyond breastfeeding, we found that consumption of fish, vegetables and fruit and berries was associated with higher neurodevelopmental scores. This relation was still present after adjusting for the child’s gender, age, parental education and duration of breastfeeding, indicating that these food items could be important contributors to neurodevelopment. Previous studies have explored relations between early diet and later development and academic achievement. Findings from the Raine cohort showed that diet at one year of age was associated with cognitive outcomes at 10 years of age [2]. Our findings demonstrate that associations between diet and child neurodevelopment can be measurable already in the first years of life. 

There may be several mechanisms through which various aspects of a healthy diet are related to neurodevelopment. First, fatty acids in fish, as well as the content of iodine, are important for brain development [18,34,35]. Further, fruits and vegetables provide a broad range of micronutrients that are necessary for brain growth and development. For instance, carotenoids and vitamin C, found in abundance in fruits and vegetables, are presumed to play important roles in brain development and functioning [36,37,38]. 

Haapala et al. found that intake of red/processed meat was inversely associated with executive functioning in 7-years old children [39]. In our sample, we found a positive association between meat intake and ASQ score. However, when we removed the intake of liver paste from the variable on meat and meat products, this positive association was no longer present. Liver paste is a popular spread, especially among young children, and most kindergartens in Norway offer liver paste as a spread alternative. Liver paste has a relatively high iron content, which is important for development, and it is often used as an example of an iron-rich food recommended for children. Further research on this observed association between intake of liver paste and total ASQ score is needed to confirm our findings. 

A significant strength of our study concerns generalisability. First, the participants were from 43 different kindergartens from four counties in different parts of Norway. Both large and small, private and public kindergartens were represented from both urban and rural areas, so it is probable that the kindergartens included in our study are representative of Norwegian kindergartens.

Second, we distributed the full version of the ASQ as published, which included pictograms and instructions to attempt every activity with the child. Some earlier studies, using the ASQ as a measure of infant development, have simplified the questionnaire, for instance using it without pictograms, without prompts to try the activity with the child, or using shortened versions with only a few questions or selected domains [40,41,42]. Valla et al. concluded that it seems important to use the correct published version [42]. We also managed to distribute the age-related questionnaire (bimonthly specific) at the relevant age by calculating each child’s age at the day of mailing the questionnaire to the parents.

Third, the study was performed in a country with high breastfeeding rates. Norway is among the countries with the highest breastfeeding rates in the world [43]. In our sample, more than 70 per cent of the children were breastfed (exclusively or partly) at 6 months of age (data not shown). 

There are also limitations to our study. First, the sample of participating parents was rather homogeneous, the majority being highly educated mothers of Norwegian ethnicity, and this may have reduced the generalisability at the individual level. However, Norwegian women are relatively highly educated, with 58.2% of women in the age of 30 to 34 years being highly educated (university or college) [44]. Other aspects, such as the geographic diversity and the diversity in size and type of kindergartens, may enhance the generalisability. 

Second, since the breastfeeding rate in this sample was high with only 15 children never being breastfed, it makes it difficult to draw conclusions regarding the associations between diet and neurodevelopment in those never breastfed. Our robustness test indicates that there might have been differences between those never and ever breastfed. However, our limited sample size suggests that such associations should be investigated further in a larger sample and in other populations. 

Third, we did not have information about the mothers IQ, which is another important determinant of neurodevelopment in children. However, we did adjust for both parents’ educational level, and although education is not the same as IQ, it is likely to represent some of the same potentials for confounding. 

Fourth, we did not have the possibility to adjust for energy intake in the models. Higher frequencies of intake could be an indicator of higher food intake in general, for example, due to a healthy appetite, larger body mass and beneficial meal routines, and the observed associations might be due to general good nourishment. 

Fifth, the results of the study are based on parents’ self-report, which may have its weaknesses. Self-reported data entail a risk of social desirability bias, both as over- and under-reporting, as well as of recall bias. There are also limitations regarding the questionnaire used to assess food intake. The questionnaire does not measure absolute food intake, only frequencies of intake. It is possible that high-frequency users consume very small amounts each time, and the opposite that low-frequency users consume larger amounts each time is possible. In our study, the mean frequency of vegetable intake was quite high (approximately three times per day). This can probably be a correct measure of vegetable frequency during the day, but the amounts eaten of each vegetable do most likely not correspond to three full vegetable portions per day, which is the recommended intake of vegetables. Data shows that Norwegian children, in general, eat fewer vegetables than is recommended: the average intake in one-year-old children is only half of the recommended intake [45]. Nevertheless, FFQs are frequently used because they are simple, quick and reliable tools compared with other more time-consuming dietary assessment methods [46]. We considered the FFQ suitable for use in our study since we primarily wanted it to measure the vegetable variety and certain types of foods eaten, as well as to rank individuals according to food intake, rather than to measure the amount of food or calories in the children’s diet. 

## 5. Conclusions

In the present study, we found an association between neurodevelopmental score, measured with the Ages and Stages Questionnaire, and the duration of breastfeeding. As one of the first studies, we also found associations between dietary factors and neurodevelopment in children as young as one year old. We found strong associations between total ASQ score and the intake of fish, fruits and vegetables in one-year-old children. Our results indicate that a healthy diet is important for neurodevelopment in young children, with measurable effects already at the age of one year. To confirm our findings, we suggest further investigation in larger samples and different populations. 

## Figures and Tables

**Table 1:** Baseline characteristics of the study population.

Variable	Value
Children registered for the study (N)	267
Answered main questionnaire on demographics, diet etc. N (%)	246 (92.1)
Mean age in months (SD)	16.7 (3.0)
Gender Female N (%)	117 (47.6)
Parents	
Mean age in years (SD)	31.2 (4.7)
Gender * Female N (%)	218 (88.6)
Parents living together (%)	94.3
Ethnicity mother born in Norway N (%)	225 (91.5)
Ethnicity father born in Norway N (%)	220 (89.4)
Mothers education N (%) Primary school Upper secondary school/High school University/College <4 years University/College >4 years	10 (4.1) 79 (32.1) 103 (41.9) 54 (22.0)
Fathers education N (%) Primary school Upper secondary school/High school University/College <4 years University/College >4 years	7 (2.8) 135 (54.9) 62 (25.2) 42 (17.1)
Duration of breastfeeding in weeks (SD)	33.3 (18.4)
Median (min-max)	36.0 (0–55)
IQR ^**^ (25th to 75th percentile)	20–52

* Gender of the parent who answered the baseline questionnaire; ** Interquartile range.

**Table 2:** Number of children assessed with each age-specific Ages and Stages Questionnaire version, number excluded due to preterm birth and mean/median total ASQ scores.

Age of ASQ Assessment	Number of Participants	Number Completed ASQ (%)	Number Born >3 Weeks Pre-Term (Excluded)	Total ScoreMean (SD)	Median (Min–Max)
ASQ	267	232 (86.9)	12	235.3 (37.4)	240 (125–300)
10 months	7	7 (100)	1	230 (47.4)	238 (150–280)
12 months	34	26 (76.5)	1	232 (38.6)	240 (160–290)
14 months	41	37 (90.2)	2	238 (39.5)	245 (135–300)
16 months	62	56 (90.3)	3	229 (37.3)	230 (140–295)
18 months	58	50 (86.2)	3	231 (35.7)	235 (140–290)
20 months	48	41 (85.4)	2	248 (28.8)	245 (160–300)
22 months	17	15 (88.2)	0	239 (48.2)	240 (125–295)

ASQ: Ages and stages questionnaire.

**Table 3:** Children’s dietary intake at baseline (*n* = 246).

Dietary Intake (Times per Week):	Mean (SD)	Median (IQR) *
Vegetables total (incl. veg as a spread)	20.3 (11.3)	18.7 (12.6–25.4)
Fresh fruits total (incl. berries, fruits as a spread)	17.0 (10.7)	15.0 (10.5–21.0)
All fruits and vegetables ^1^	40.5 (19.9)	37.4 (26.2–50.2)
Fish (lean and fatty)	2.1 (1.3)	2.0 (1–3)
Fish and fish products (incl. fish as a spread)	5.9 (3.7)	5.5 (3.1–7.6)
Unprocessed meat (red meat and poultry)	2.2 (1.8)	2.0 (1.0–3.0)
All meat and meat products ^2^	10.1 (4.4)	9.5 (7.1–12.5)
All meat and meat products minus liver paste	6.3 (3.6)	5.5 (4.0–7.5)
Liver paste (spread)	3.8 (2.2)	3.5 (3.5–5.5)
Wholegrain products ^3^	11.7 (5.0)	11.3 (8.5–14.1)
Typical sugary foods ^4^	3.9 (3.7)	2.7 (1.3–5.6)

* IQR: Interquartile range (25th–75th percentile); ^1^ Fresh fruits, berries, vegetables, fresh potatoes, legumes and unsalted nuts; ^2^ Unprocessed red and white meat, minced meat, sausages, includes meat as a spread (ham, cold cuts, liver paste)^; 3^ Wholegrain bread and crispbread, oatmeal porridge, oatmeal and wholegrain müsli; ^4^ sweets, candy, snacks, ice cream, biscuits, sugary drinks and chocolate spread.

**Table 4:** Linear regression on associations between dietary factors and ASQ total score.

Dietary Factor	Unadjusted Model			Adjusted Model *			Adjusted Model Incl. Breastfeeding **		
	β	95% CI	*p*-Value	β	95% CI	*p*-Value	β	95% CI	*p*-Value
Duration of breastfeeding (weeks)	0.39	0.11–0.67	0.007	0.42	0.14–0.71	0.004	n.a ***	n.a ***	n.a ***
Fresh fruits and berries (times/week)	0.51	0.05–0.97	0.030	0.50	0.04–0.96	0.033	0.45	0.00–0.90	0.050
Vegetables (times/week)	0.94	0.50–1.37	<0.001	0.90	0.47–1.34	<0.001	0.88	0.45–1.31	<0.001
All fruits and vegetables (incl. potato, legumes) (times/week)	0.46	0.21–0.70	<0.001	0.44	0.20–0.69	<0.001	0.42	0.18–0.66	0.001
Fish (lean and fatty) (times/week)	4.21	0.24–8.19	0.038	4.28	0.36–8.21	0.033	3.90	0.02–7.76	0.049
All fish and fish products (incl. spread) (times/week)	1.79	0.47–3.11	0.008	1.85	0.54–3.15	0.006	1.70	0.41–2.99	0.010
Unprocessed red and white meat (times/week)	0.72	−2.02–3.45	0.605	1.17	−1.55–3.88	0.398	1.61	−1.07–4.29	0.237
All meat and meat products (times/week)	1.12	0.00–2.25	0.050	1.13	0.01–2.25	0.047	1.17	0.07–2.27	0.037
All meat and meat products minus liver paste (times/week)	0.86	−0.53–2.26	0.223	0.82	−0.58–2.22	0.247	0.94	−0.44–2.31	0.179
Liver paste (times/week)	2.30	0.02–4.58	0.049	2.44	0.18–4.70	0.034	2.29	0.07–4.51	0.044
Wholegrain products (times/week)	1.24	0.24–2.24	0.016	1.07	0.05–2.09	0.039	0.88	−0.13–1.89	0.088
Sugary foods (times/week)	1.23	−0.12–2.59	0.075	1.16	−0.26–2.58	0.109	1.15	−0.25–2.54	0.107

* Adjusted for child age, child gender, maternal and paternal education; ** Adjusted for child age, child gender, maternal and paternal education and duration of breastfeeding. *** Not applicable since the variable Duration of breastfeeding (weeks) is a covariate that is adjusted for in this model.
